# Slaughtered pigs as reservoirs of One Health-relevant antimicrobial resistance genes in *Klebsiella pneumoniae* from Brazil

**DOI:** 10.3389/fmicb.2026.1927798

**Published:** 2026-09-09

**Authors:** Gabriel Rangel Azevedo, Agostinho Sérgio Scofano, Daniele Soares Fialho, Gisllany Alves Costa, Maria Helena Cosendey de Aquino, Dayse Lima da Costa Abreu, Elmiro Rosendo do Nascimento, Thomas Salles Dias

**Affiliations:** 1 Departamento de Saúde Coletiva Veterinária e Saúde Pública, Universidade Federal Fluminense, Rio de Janeiro, Brazil; 2 Instituto de Defesa Agropecuária e Florestal do Espírito Santo, Vitória, Espírito Santo, Brazil

**Keywords:** antimicrobial resistance, CTX-M, food safety, *Klebsiella pneumoniae*, multidrug resistance, One Health, swine

## Abstract

**Introduction:**

*Klebsiella pneumoniae* is an important opportunistic pathogen and a recognized reservoir of antimicrobial resistance (AMR) determinants. However, information on AMR profiles and genomic characteristics of *Klebsiella* circulating in Brazilian swine production remains limited. This study investigated the occurrence, antimicrobial susceptibility, resistance determinants, and genomic characteristics of *Klebsiella* spp. recovered from pigs at slaughter in southeastern Brazil.

**Methods:**

Rectal swabs were collected from 100 pigs at slaughter, and *Klebsiella* isolates were identified by culture and PCR. Antimicrobial susceptibility was determined by disk diffusion and broth microdilution for colistin. AMR genes were investigated by PCR, and four representative multidrug-resistant (MDR) *K. pneumoniae* isolates carrying multiple β-lactam, quinolone, and tetracycline resistance genes were further characterized by whole-genome sequencing and comparative genomic analysis.

**Results:**

Fifty-three *Klebsiella* isolates were recovered, including 47 *K. pneumoniae*, four *Klebsiella oxytoca*, one *Klebsiella quasipneumoniae*, and one *Klebsiella* spp. High frequencies of non-susceptibility were observed for tetracycline (86.8%), ciprofloxacin (77.4%), and amoxicillin–clavulanate (67.9%), while 34 isolates (64.2%) were classified as MDR. Three colistin-resistant isolates carried *mcr-1*. The most frequently detected AMR genes were *bla*_*SHV*_, *bla*_*TEM*_, *tetA*, *qnrS*, and *oqxAB*. Whole-genome sequencing identified two genomic lineages, ST45 and ST1027, differing in resistance gene content and capsular and O-antigen loci. All sequenced isolates carried the acquired siderophore loci *ybt* and *iuc*, indicating the coexistence of acquired virulence-associated loci and multiple AMR genes.

**Discussion:**

These findings demonstrate a high occurrence of MDR *K. pneumoniae* carrying clinically important resistance determinants in pigs at slaughter and reveal the circulation of distinct genomic lineages harboring acquired virulence-associated loci. The results suggest that swine production systems may contribute to the maintenance and potential dissemination of One Health-relevant *K. pneumoniae* and highlight the importance of integrated genomic surveillance to support antimicrobial stewardship and food safety.

## 1 Introduction

Pork remains one of the most widely consumed sources of animal protein worldwide and is expected to contribute significantly to the projected growth in global meat demand through 2030 (Organisation for Economic Co-operation and Development [OECD], and Food and Agricultural Organization [FAO], 2021). In this context, Brazil stands out as the world’s fourth-largest pork producer and third-largest pork exporter, reinforcing the economic and social relevance of swine production (Associação Brasileira de Proteína Animal [ABPA], 2026; United States Department of Agriculture Foreign Agricultural Service [USDA-FAS], 2026). Increasing national and international market demands have driven the modernization of swine production systems through the continuous adoption of new technologies and improved management practices. In this context, sustainable pig farming depends on the rational use of resources combined with strategies that promote animal health, welfare, and production efficiency (Fischer et al., 2019; Albernaz-Gonçalves et al., 2022).

Antimicrobial use plays a central role in animal production, being employed for therapeutic, prophylactic, and metaphylactic purposes, as well as for growth promotion (Stella et al., 2020). However, the inappropriate use and overuse of antimicrobials in human medicine and food production have contributed to the emergence and dissemination of antimicrobial resistance (AMR), which threatens the effectiveness of modern medicine and global public health responses to infectious diseases (World Health Organization [WHO], 2015). According to the World Health Organization (WHO) One Health concept, the health of humans, animals, plants, and ecosystems are closely linked and interdependent, making AMR a challenge that requires integrated and multisectoral approaches (World Health Organization [WHO], 2023). Exposure to antimicrobial-resistant bacteria and resistance determinants from food-producing animals may occur through the consumption of contaminated food products, highlighting the role of the food chain in the dissemination of resistant bacteria and resistance determinants (Chang et al., 2015; Samtiya et al., 2022).

Among the microorganisms of concern in this context, *Klebsiella* spp. are Gram-negative, non-motile, rod-shaped facultative anaerobes of the Enterobacteriaceae family that are widely distributed in mammals, insects, and environmental sources, including soil, water, and vegetation (Brisse et al., 2006; Araújo et al., 2025). *Klebsiella pneumoniae* is a clinically important opportunistic pathogen frequently implicated in both nosocomial and community-acquired infections (Podschun and Ullmann, 1998; Navon-Venezia et al., 2017). Furthermore, the relevance of *K. pneumoniae* is underscored by the WHO Bacterial Priority Pathogens List (World Health Organization [WHO], 2024b), which classifies carbapenem- and third-generation cephalosporin-resistant Enterobacterales in the critical category of bacterial pathogens that pose the highest threat to public health.

The concern surrounding *K. pneumoniae* is further amplified by the increasing emergence of multidrug-resistant (MDR) strains, which significantly limit therapeutic options (Navon-Venezia et al., 2017; Huy, 2024). Horizontal gene transfer mediated by mobile genetic elements plays a major role in the dissemination of AMR and virulence determinants in *K. pneumoniae*, a species currently divided into distinct pathotypes, including classical *K. pneumoniae* (cKp), commonly associated with opportunistic infections, and hypervirulent *K. pneumoniae* (hvKp), capable of causing severe infections even in otherwise healthy individuals (Shon et al., 2013; Russo and Marr, 2019).

In addition to their clinical importance, *Klebsiella* spp. associated with AMR have also been recovered from a wide variety of food products, including raw meat, raw vegetables, and ready-to-eat foods, highlighting their potential relevance to food safety (Hartantyo et al., 2020; Theocharidi et al., 2022; Crippa et al., 2023). The presence of *Klebsiella* spp. in the food chain has become an increasing concern, as gastrointestinal colonization may precede infection while also providing conditions that may facilitate the dissemination of AMR and virulence determinants through horizontal gene transfer (Martin and Bachman, 2018; Crippa et al., 2023).

In animal production, particularly in swine, *Klebsiella* spp. have been associated with pneumonia, mastitis, septicemia, and other infectious disorders, while pigs may also act as reservoirs of antimicrobial-resistant strains (Bidewell et al., 2018; Samanta et al., 2018; Sofiana et al., 2020; Ribeiro et al., 2022). Furthermore, these bacteria may spread during different stages of food production, including slaughter and processing, leading to contamination of food products and potential consumer exposure (Kurittu et al., 2021). Therefore, the present study aimed to investigate the occurrence of *Klebsiella* spp. in the swine production chain in the Southeast region of Brazil and to characterize their phenotypic and genotypic AMR profiles.

## 2 Materials and methods

### 2.1 Study design and sample collection

This cross-sectional study was conducted using convenience sampling of slaughter batches available during the sampling period. A total of 100 rectal swab samples were collected from pigs reared under intensive production systems in the state of Espírito Santo, Brazil. The sampled animals originated from five production batches intended for slaughter, each batch corresponding to a different farm. A total of 22 swabs were collected from four batches, whereas 12 swabs were collected from one batch. The farms were selected based on access to slaughter batches available at the time of sampling and were not intended to represent all swine farms in the state. Therefore, the results should be interpreted as the occurrence and antimicrobial resistance profile of *Klebsiella* spp. among the sampled slaughter pigs, rather than as a population-level prevalence estimate.

One rectal swab was collected from each animal immediately prior to slaughter in order to assess fecal carriage as a potential source of carcass contamination during processing. Sterile rectal swabs were placed in Cary-Blair transport medium and transported to the laboratory under appropriate conditions for microbiological analysis.

The animals received antimicrobial agents via feed supplementation in six out of seven production phases (29–150 days of age), according to routine farm management practices. During the pre-starter phase (29–35 days), gentamicin (aminoglycoside) and amoxicillin (β-lactam) were administered. In the starter phase I (36–50 days), amoxicillin and tilmicosin (macrolide) were used, and from 51 to 70 days animals continued to receive amoxicillin.

In the subsequent phases, a broader range of antimicrobials was introduced. Between 71 and 95 days, animals were exposed to chlortetracycline (tetracycline), sulfamethoxazole–trimethoprim (sulfonamide combination), and citric acid. From 96 to 120 days, doxycycline (tetracycline) and tiamulin (pleuromutilin) were administered, whereas florfenicol (phenicol) was included in the feed during the 121–145-days phase. No antimicrobial supplementation was provided during the final phase (146–150 days).

All antimicrobial treatments followed recommended withdrawal periods prior to slaughter, ranging from 4 to 15 days depending on the compound, in accordance with veterinary guidelines.

### 2.2 Bacterial isolation and phenotypic identification

Isolation of *Klebsiella* spp. from rectal swab samples was performed using a culture-based approach adapted from previously described protocols (Van Kregten et al., 1984; Passet and Brisse, 2015).

Briefly, samples were subjected to a pre-enrichment step in Brain Heart Infusion (BHI) broth and incubated at 37 °C for 18–24 h. Subsequently, aliquots were streaked onto Simmons citrate agar supplemented with inositol (SCAi), a selective and differential medium that allows presumptive identification of *Klebsiella* spp. based on inositol fermentation and citrate utilization. Plates were incubated at 37 °C for 24–48 h.

Up to five colonies exhibiting typical morphology (yellow, mucoid, dome-shaped) were selected from each SCAi plate and subjected to biochemical identification using Triple Sugar Iron (TSI) agar and Sulfide-Indole-Motility (SIM) medium, following standard microbiological procedures.

### 2.3 Hypermucoviscosity assessment

The hypermucoviscous phenotype was evaluated by the string test as previously described by Fang et al. (2004). Isolates were cultured overnight at 37 °C on Mueller-Hinton agar plates. A bacteriology inoculation loop was used to stretch a colony upward, and the formation of a viscous string was observed. Isolates were considered positive for the hypermucoviscous phenotype when a mucoviscous string measuring >5 mm in length was formed.

### 2.4 Molecular identification

Confirmation of the *Klebsiella* genus was performed by polymerase chain reaction (PCR) targeting the *gyrA* gene (Brisse and Verhoef, 2001). Differentiation of *K. pneumoniae*, *Klebsiella variicola*, and *Klebsiella quasipneumoniae* was performed by amplification of the *bla*_*SHV*_, *bla*_*LEN*_, and *bla*_*OKP*_ genes, respectively, according to Fonseca et al. (2017), whereas *Klebsiella oxytoca* was identified by amplification of the *pehX* gene (Kovtunovych et al., 2003). PCR conditions and primer sequences followed the published protocol. Primer sequences are provided in Supplementary Table 1.

### 2.5 Antimicrobial susceptibility testing

Antimicrobial susceptibility testing was performed by the disk diffusion method on Mueller-Hinton agar (HiMedia, India), in accordance with the guidelines of the Clinical and Laboratory Standards Institute (CLSI) (2025). Bacterial suspensions were adjusted to a 0.5 McFarland standard and inoculated onto Mueller-Hinton agar plates. The following antimicrobial agents were tested: tetracycline (30 μg), ciprofloxacin (5 μg), amoxicillin–clavulanate (20/10 μg), cefoxitin (30 μg), cefotaxime (30 μg), ceftazidime (30 μg), cefepime (30 μg), imipenem (10 μg), and meropenem (10 μg). Plates were incubated at 37 °C for 18–24 h. Colistin susceptibility was determined by the broth microdilution method. *Escherichia coli* ATCC 25922 was used as a quality control strain. MDR isolates were defined as those exhibiting acquired non-susceptibility to at least one agent in three or more antimicrobial categories, according to the criteria proposed by Magiorakos et al. (2012).

### 2.6 Detection of AMR genes

Detection of AMR genes was performed by PCR. Determinants associated with resistance to tetracyclines (*tetA*, *tetB*, *tetC*, *tetD*, *tetE*, *tetG*) (Ng et al., 2001), quinolones (*qnrA*, *qnrB*, *qnrC*, *qnrD*, *qnrS*, *oqxAB*, *qepA*, *aac(6*′*)-Ib-cr*) (Park et al., 2006; Yamane et al., 2008; Chen et al., 2012; Kraychete et al., 2016), and colistin (*mcr-1* to *mcr-10*) (Rebelo et al., 2018; Borowiak et al., 2020; Lei et al., 2020) were investigated only in isolates classified as non-susceptible in the antimicrobial susceptibility testing, whereas β-lactam resistance genes (*bla*_*SHV*_, *bla*_*CTX–M*_, *bla*_*TEM*_, *bla*_*CMY*_) (Koeck et al., 1997; Monstein et al., 2007) were screened in all *Klebsiella* spp. isolates. Primer sequences are provided in Supplementary Table 2.

Polymerase chain reaction reactions were carried out in a final volume of 25 μL containing 1× buffer, 0.2 μM of each dNTP, 0.2 μM of each primer, 1 U of Taq DNA polymerase (Promega, Brazil), and 100 ng of template DNA.

Amplified products were separated by electrophoresis on 1.5% agarose gel prepared in Tris-acetate-EDTA (TAE) buffer and stained with ethidium bromide (0.5 μg/mL). Electrophoresis was conducted at 90 V for 40 min.

Amplicons were visualized under ultraviolet light using a transilluminator, and images were captured with the L-Pix documentation system (Loccus, Brazil).

### 2.7 Whole genome sequencing

To further characterize the genetic basis of antimicrobial resistance, four MDR isolates carrying *bla*_*SHV*_, *bla*_*TEM*_, and *bla*_*CTX–M*_, in addition to genes associated with quinolone and tetracycline resistance, were selected for whole-genome sequencing (WGS).

Genomic libraries were prepared using the NEBNext^®^ Ultra II DNA Library Prep Kit (Illumina, Inc., Cambridge, UK), and sequencing was performed on the Illumina NovaSeq platform using paired-end reads (2 × 150 bp).

Raw reads were quality-checked using FastQC, and genome assembly and annotation were conducted using the Bacterial and Viral Bioinformatics Resource Center (BV-BRC). Identification of AMR genes and chromosomal mutations was performed using pipelines available at the Center for Genomic Epidemiology (CGE). Plasmid replicon screening was performed by BLASTn search against the PlasmidFinder database, using thresholds of ≥95% nucleotide identity and ≥60% query coverage. Detected replicons were interpreted as putative plasmid-associated sequences. Because the genomes were generated using short-read sequencing, complete plasmid reconstruction and definitive localization of antimicrobial resistance genes were not performed. Multi-locus sequence typing (MLST) was determined using the PubMLST platform.

### 2.8 Phylogenetic analysis

A comparative genomic analysis was performed using four *K. pneumoniae* isolates from this study and 16 publicly available human-associated *K. pneumoniae* genomes. The isolates originally identified as KB6, KB21, KB34, and KB38 were renamed MSV_UFF_KB6, MSV_UFF_KB21, MSV_UFF_KB34, and MSV_UFF_KB38 for the genomic analyzes and are available under the BioSample accessions SAMN61231285, SAMN61231286, SAMN61231287, and SAMN61231288, respectively.

Publicly available human-associated *K. pneumoniae* genomes were selected to provide a focused comparative context for the sequence types detected among the sequenced swine isolates. After MLST analysis identified ST45 and ST1027 among the isolates from this study, NCBI databases were searched for publicly available human-associated genomes belonging to these same STs. Genomes were included when sequence data were available and when metadata supported human association and compatible species/ST assignment. This search resulted in the inclusion of 15 human-associated ST45 genomes and the only human-associated ST1027 genome. Therefore, the comparative dataset was designed to contextualize the swine isolates within publicly available human-associated genomes of the same STs, rather than to represent the full global diversity of *K. pneumoniae*.

Public genome assemblies from ST45 and ST1027 were retrieved using NCBI Datasets. Since no assembled genome was available for the human ST1027 isolate SRR10882645 through NCBI Assembly, the raw FASTQ file was downloaded and *de novo* assembled using SPAdes^1^. Species identification, MLST, antimicrobial resistance gene detection, virulence-associated locus screening and K/O locus typing were performed using Kleborate^2^ with the preset for the *K. pneumoniae* species complex. Whole-genome distances were estimated using Mash^3^.

Whole-genome relatedness was assessed using Mash. Genome sketches were generated from all assemblies, and pairwise Mash distances were calculated among genomes. The resulting distance matrix was converted into a Newick-formatted tree using a custom Python script based on hierarchical clustering. The tree was visualized and annotated using iTOL.

## 3 Results

Following biochemical characterization and PCR-based molecular identification of the genus and species, 53 *Klebsiella* spp. isolates were obtained from the analyzed samples. Of these, 47 were identified as *K. pneumoniae*, four as *K. oxytoca*, one as *K. quasipneumoniae*, and one as *Klebsiella* spp. All samples yielding *Klebsiella* isolates harbored a single *Klebsiella* species, except for one sample in which two distinct species were recovered, identified as *K. pneumoniae* and *Klebsiella* spp. Seven *K. pneumoniae* isolates, each recovered from a different sample, exhibited a hypermucoviscous phenotype.

Antimicrobial susceptibility testing demonstrated a high prevalence of non-susceptibility among the isolates (Table 1). Four isolates were classified as non-susceptible to colistin based on minimum inhibitory concentration (MIC) assays, with MIC values of 16 μg/mL (*n* = 1), 8 μg/mL (*n* = 1), and 4 μg/mL (*n* = 2). Among β-lactams, non-susceptibility was most frequently observed for amoxicillin–clavulanate (*n* = 36), followed by cefotaxime (*n* = 15), cefepime (*n* = 10), ceftazidime (*n* = 5), and cefoxitin (*n* = 3), whereas no non-susceptibility was detected for meropenem or imipenem. Overall, 40 isolates were non-susceptible to at least one β-lactam agent. Regarding quinolones, 41 isolates were non-susceptible to ciprofloxacin. Among tetracyclines, non-susceptibility to tetracycline was observed in 46 isolates. Notably, β-lactams and tetracyclines were among the antimicrobial classes administered during the production cycle.

**TABLE 1:** Frequency of antimicrobial non-susceptibility in *Klebsiella* spp. isolates recovered from pigs at slaughter in the Southeast region of Brazil.

Antimicrobial class	Antimicrobial	Non-susceptible isolates (*n*)	Frequency (%)
**Polymyxin**	Colistin	4	7.5
**β -lactams**	Amoxicillin–clavulanate	36	67.9
	Cefoxitin	3	5.7
	Cefotaxime	15	28.3
	Ceftazidime	5	9.4
	Cefepime	10	18.9
	Imipenem	0	0
	Meropenem	0	0
**Quinolones**	Ciprofloxacin	41	77.4
**Tetracyclines**	Tetracycline	46	86.8

A total of 34 (64.2%) isolates were classified as MDR. Among these, four isolates concurrently exhibited MDR and hypermucoviscous phenotypes. In contrast, six isolates were fully susceptible to all antimicrobials tested and consisted of all four *K. oxytoca* isolates and two *K. pneumoniae* isolates.

Molecular analyzes revealed the presence of multiple AMR determinants (Table 2). Concerning colistin resistance, three isolates harbored the *mcr-1* gene, whereas *mcr-2* to *mcr-10* were not detected. One isolate exhibited phenotypic resistance to colistin in the absence of the investigated *mcr* genes.

**TABLE 2:** Frequency of AMR determinants identified in *Klebsiella* spp. isolates recovered from pigs at slaughter in the Southeast region of Brazil.

Antimicrobial class	Resistance determinant	*n*/*N*	Frequency (%)
**Colistin**	*mcr-1*	3/4	75.0
	*mcr-2–mcr-10*	0/4	0.0
**β -lactams**	*bla*_SHV_	47/53	88.7
	*bla*_TEM_	42/53	79.2
	*bla*_CTX–M_	14/53	26.4
	*bla*_CMY_	0/53	0.0
**Quinolones**	*qnrS*	34/41	82.9
	*oqxAB*	34/41	82.9
	*aac(6*′*)-Ib-cr*	22/41	53.7
	*qnrB*	2/41	4.9
	*qnrA*/*qnrC*/*qnrD*/*qepA*	0/41	0.0
**Tetracyclines**	*tetA*	43/46	93.5
	*tetB*	8/46	17.4
	*tetD*	1/46	2.2
	*tetC*/*tetE*/*tetG*	0/46	0.0

*n*, number of isolates positive for the respective resistance determinant; *N*, number of isolates screened for the corresponding determinant. Genes associated with colistin, quinolone, and tetracycline resistance were investigated only in phenotypically non-susceptible isolates, whereas β-lactam resistance genes were screened in all *Klebsiella* spp. isolates.

With respect to β-lactam resistance genes, *bla*_SHV_ was detected in 47 isolates, *bla*_TEM_ in 42, and *bla*_CTX–M_ in 14 isolates, while *bla*_CMY_ was not identified. Notably, ten isolates co-harbored *bla*_SHV_, *bla*_CTX–M_, and *bla*_TEM_ genes.

Regarding quinolone resistance determinants, *qnrS* was identified in 34 isolates, the *oqxAB* operon was detected in 34 isolates, and *aac(6*′*)-Ib-cr* was present in 22 isolates. The *qnrB* gene was detected in two isolates, whereas *qnrA*, *qnrC*, *qnrD*, and *qepA* were not identified.

Among tetracycline resistance genes, *tetA* was the most prevalent (*n* = 43), followed by *tetB* (*n* = 8) and *tetD* (*n* = 1), while *tetC*, *tetE*, and *tetG* were not detected. The relationship between antimicrobial resistance phenotypes and detected resistance determinants is summarized in Table 3.

**TABLE 3:** Association between antimicrobial resistance phenotypes and detected resistance determinants in *Klebsiella* spp. isolates recovered from pigs at slaughter in the Southeast region of Brazil.

Antimicrobial class	Isolateso n-susceptible to ≥1 antimicrobial agent within the class (*n*)	Resistance determinants detected	Isolates carrying ≥1 determinant associated with the class (*n*)
**Colistin**	4	*mcr-1*	3
**β -lactams**	40	*bla*_CTX–M_, *bla*_SHV_, *bla*_TEM_	53
**Quinolones**	41	*qnrB*, *qnrS*, *oqxAB*, *aac(6*′*)-Ib-cr*	40
**Tetracycline**	46	*tetA*, *tetB*, *tetD*	46

Genes associated with colistin, quinolone, and tetracycline resistance were investigated only in phenotypically non-susceptible isolates, whereas β-lactam resistance genes were screened in all *Klebsiella* spp. isolates.

High-quality sequencing data were obtained for all four selected *K. pneumoniae* isolates. The four genomes generated between 9,927,759 and 11,078,435 reads, corresponding to 2.98–3.32 Gb of sequencing data per isolate. Sequencing quality was high, with Q20 values ranging from 99.01% to 99.07% and Q30 values from 95.15% to 95.40%. The raw GC content ranged from 57.13% to 57.31%. Genome assemblies ranged from 5,433,734 to 5,723,483 bp, with GC contents between 56.98% and 57.24%. The number of contigs ranged from 73 to 124, and N50 values ranged from 233,891 to 296,421 bp. Mean sequencing depth ranged from 265.41× to 549.41×. These assembly metrics supported downstream genome-based analyzes, including species confirmation, MLST, K/O locus typing, virulence-associated locus screening, and antimicrobial resistance gene detection. Detailed sequencing quality metrics and genome assembly statistics are provided in Supplementary Table 3.

Whole-genome analysis of the four selected *K. pneumoniae* isolates confirmed all genomes as *K. pneumoniae* with strong species matches. Genome assemblies ranged from 103 to 124 contigs, with total genome sizes between 5,670,243 and 5,723,483 bp. Multilocus sequence typing revealed two sequence types among the sequenced isolates: MSV_UFF_KB6 and MSV_UFF_KB21 belonged to ST1027, whereas MSV_UFF_KB34 and MSV_UFF_KB38 belonged to ST45. The ST1027 isolates shared the same allelic profile, *gapA*-2, *infB*-1, *mdh*-1, *pgi*-1, *phoE*-9, *rpoB*-4, and *tonB*-14, while ST45 isolates presented the profile *gapA*-2, *infB*-1, *mdh*-1, *pgi*-6, *phoE*-7, *rpoB*-1, and *tonB*-12.

All sequenced isolates carried virulence-associated loci related to iron acquisition. The yersiniabactin locus was identified as *ybt*10 associated with ICEKp4 in all four genomes. In addition, all isolates harbored the aerobactin locus *iuc*3. No colibactin, salmochelin, or *rmpADC* loci were detected. The four isolates presented a Kleborate virulence score of 4, reflecting the presence of acquired virulence-associated determinants, mainly related to siderophore-mediated iron acquisition.

**FIGURE 1:**
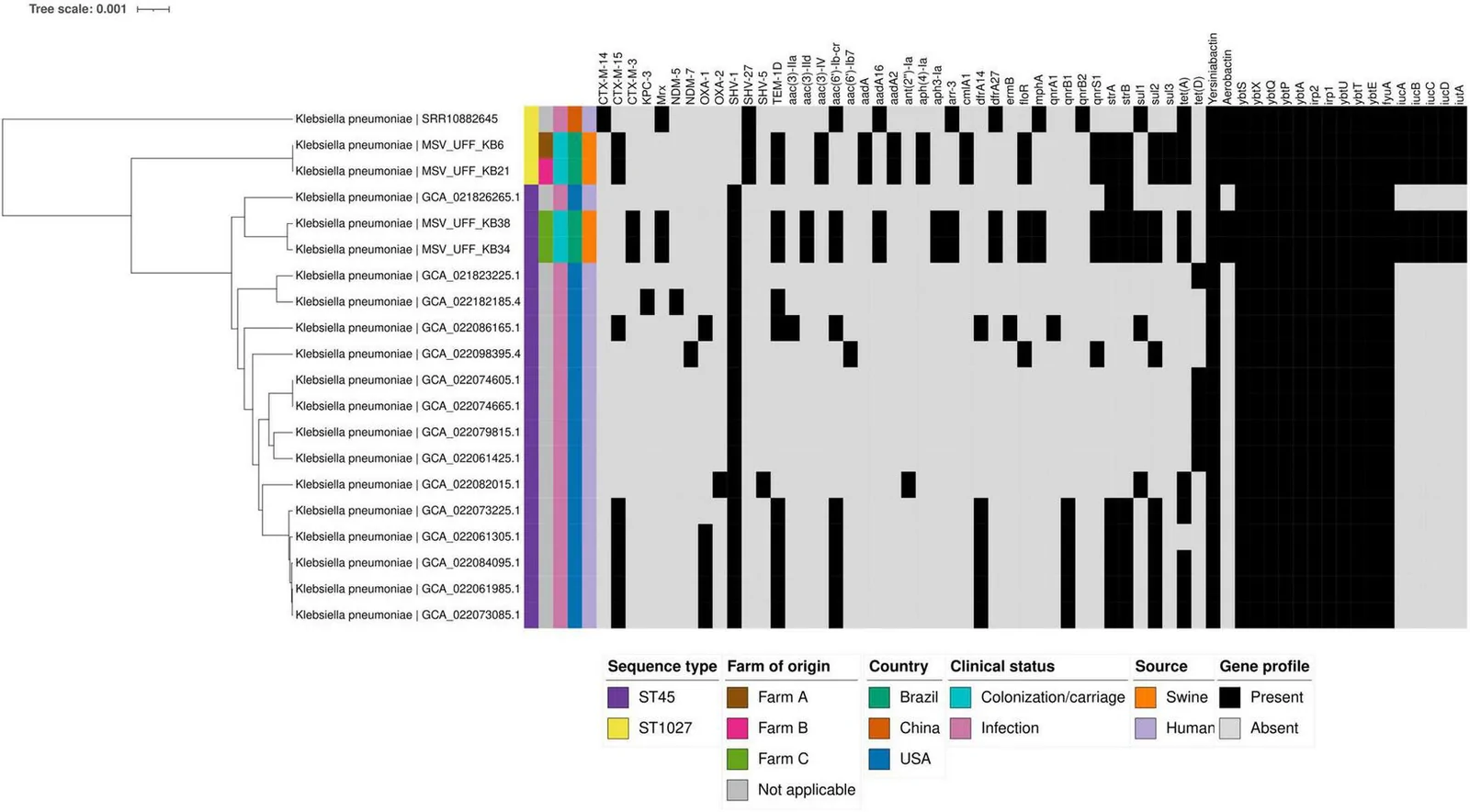
Phylogenetic tree and heatmap showing the distribution of antimicrobial resistance and virulence-associated genes among *K. pneumoniae* isolates. Black boxes indicate the presence of genes, whereas gray boxes indicate their absence. Color-coded annotation strips indicate sequence type, farm of origin, country, clinical status, and source. For farm origin, MSV_UFF_KB6, MSV_UFF_KB21, and MSV_UFF_KB34/MSV_UFF_KB38 were assigned to different farms, with MSV_UFF_KB34 and MSV_UFF_KB38 originating from the same farm. Public human-associated genomes were marked as not applicable for farm origin. The phylogenetic tree was generated based on whole-genome sequence data.

Whole-genome analysis also allowed refinement of antimicrobial resistance gene profiles. MSV_UFF_KB6 and MSV_UFF_KB21 carried *bla*_CTX–M–15_, *bla*_TEM–1D_, *bla*_SHV–27_, *qnrS1*, *tetA*, *sul2*, *sul3*, *cmlA1*, *floR*, and aminoglycoside resistance genes including *aac(3)-IV*, *aadA2*, *aadA*, *aph(4)-Ia*, *strA*, and *strB*. These two isolates presented resistance determinants distributed across seven antimicrobial classes, totaling 14 resistance genes. In contrast, MSV_UFF_KB34 and MSV_UFF_KB38 carried *bla*_CTX–M–3_, *bla*_TEM–1D_, *bla*_SHV–1_, *qnrS1*, *aac(6*′*)-Ib-cr*, *aac(3)-IId*, *aadA16*, *aph3-Ia*, *strA*, *strB*, *mphA*, *floR*, *arr-3*, *sul1*, *sul2*, *tetA*, and *dfrA27*, totaling 17 resistance genes distributed across ten antimicrobial classes (Figure 1).

The resistance genes detected by WGS were consistent with the phenotypic and PCR-based findings. All four sequenced isolates were confirmed as *K. pneumoniae* and carried *bla*_SHV_ alleles, supporting the previous PCR-based species assignment. In addition, the detection of *bla*_CTX–M_, *bla*_TEM_, and *bla*_SHV_ variants was consistent with β-lactam non-susceptibility, while the presence of *qnrS1* and, in ST45 isolates, *aac(6*′*)-Ib-cr*, supported the quinolone non-susceptibility phenotype. Similarly, the detection of *tetA* in all four genomes was consistent with tetracycline non-susceptibility. Conversely, no carbapenemase genes were detected, which was consistent with the absence of phenotypic resistance to imipenem and meropenem in the isolate collection.

Plasmid replicon screening identified putative plasmid-associated replicons in all four sequenced *K. pneumoniae* isolates. The ST45 isolates MSV_UFF_KB34 and MSV_UFF_KB38 carried IncFII(K), IncFIB(K), IncQ1, ColRNAI, and repB(R1701) replicons. The ST1027 isolates MSV_UFF_KB6 and MSV_UFF_KB21 carried IncFII(K), IncFIB(AP001918), IncFIB(pKPHS1), ColRNAI, and repB(R1701). These findings indicate the presence of plasmid-related sequences in the selected MDR isolates. However, the genomic location of antimicrobial resistance genes could not be confirmed using short-read sequencing alone. Detailed plasmid replicon profiles are provided in Supplementary Table 4.

Capsular and O-antigen typing also differed between the two genomic groups. MSV_UFF_KB6 and MSV_UFF_KB21 were assigned to KL20/K20 and O3αβ, whereas MSV_UFF_KB34 and MSV_UFF_KB38 were assigned to KL43/K43 and O2α. These results indicate that the sequenced isolates represented two distinct genomic profiles: an ST1027/K20/O3αβ group represented by MSV_UFF_KB6 and MSV_UFF_KB21, and an ST45/K43/O2α group represented by MSV_UFF_KB34 and MSV_UFF_KB38.

## 4 Discussion

Our study demonstrates a high occurrence of *Klebsiella* spp., particularly *K. pneumoniae*, in rectal swabs from pigs at slaughter, suggesting that swine may contribute to the maintenance and dissemination of this opportunistic pathogen within the food production chain. From a food safety perspective, the detection of *Klebsiella* spp. in fecal samples collected at slaughter indicates a potential source of carcass contamination during processing. Considering the gastrointestinal tract as an important reservoir for antimicrobial-resistant bacteria, the presence of MDR *Klebsiella* spp. may contribute to human exposure through contaminated meat products and cross-contamination events along the food production chain (Wareth and Neubauer, 2021; Conceição et al., 2023).

The high frequency of AMR especially to β-lactams ciprofloxacin, and tetracycline, corroborates the current understanding of *K. pneumoniae* as a major pathogen of concern in the context of increasing AMR (Asokan et al., 2025). This pattern likely reflects the selective pressure imposed by antimicrobial use throughout the production cycle. The use of tetracyclines in later stages and the routine administration of amoxicillin in early phases may have contributed to the elevated levels of non-susceptibility observed. Comparable resistance profiles were reported by Yang et al. (2019) in China (Henan Province), where high frequencies of resistance to tetracycline and amoxicillin/clavulanic acid were observed among swine-derived *K. pneumoniae* isolates.

In contrast, the detection of non-susceptibility to quinolones and third- and fourth-generation cephalosporins is of particular concern, as these antimicrobials were not reported as part of the production regimen. These results suggest that resistance may be maintained through mechanisms such as co-selection and, potentially, horizontal gene transfer involving mobile genetic elements carrying multiple resistance determinants (Zdarska et al., 2026). Given their classification by the World Health Organization [WHO] (2024a) as highest priority critically important antimicrobials, the occurrence of non-susceptibility to these agents in isolates from food-producing animals raises significant public health concerns. Similar findings have been reported in *K. pneumoniae* isolates from pigs and other livestock production systems (Samanta et al., 2018; Kot and Witeska, 2024). Although no resistance to carbapenems was detected, which is consistent with their non-use in commercial livestock production (World Health Organization [WHO], 2024a; World Organisation for Animal Health [WOAH], 2025), the occurrence of non-susceptibility to cephalosporins and amoxicillin–clavulanate reinforces concerns regarding the circulation of β-lactam-resistant bacteria in food-producing animals.

The identification of colistin-resistant isolates is particularly noteworthy, since colistin is considered a last-resort antimicrobial for the treatment of MDR Gram-negative infections in humans (Hussein et al., 2021). The *mcr-1* gene, the first plasmid-mediated colistin resistance determinant described and widely disseminated among food-producing animals, was detected in three isolates, consistent with the recognized role of plasmid-mediated mechanisms in the dissemination of clinically relevant AMR determinants (Liu et al., 2016; Shen et al., 2020). Similarly, Phetburom et al. (2021) reported the detection of *mcr-1* in a colistin non-susceptible *K. pneumoniae* complex isolate recovered from pig carcasses at slaughter in Thailand and further identified *mcr-7* and *mcr-8*, which were not detected in the present study. Moreover, one isolate exhibited phenotypic resistance to colistin despite the absence of the investigated *mcr* genes. Besides plasmid-mediated resistance, colistin resistance in *K. pneumoniae* may arise through chromosomal mechanisms involving *mgrB*, the phoPQ and pmrAB two-component regulatory systems, and the *crrAB* operon. These mechanisms promote lipid A modifications, mainly through the addition of 4-amino-4-deoxy-L-arabinose (L-Ara4N) and phosphoethanolamine (pEtN), reducing the negative charge of the outer membrane and consequently decreasing the interaction of polymyxins with the bacterial surface (Olaitan et al., 2014; Poirel et al., 2017). Additional regulatory pathways, such as RamA, have also been associated with reduced polymyxin susceptibility (Poirel et al., 2017). Because these chromosomal mechanisms were not investigated in the present study, the genetic basis of colistin resistance in the *mcr*-negative isolate remains undetermined.

A high proportion of the isolates were classified as MDR, reinforcing the role of swine as reservoirs of antimicrobial-resistant bacteria. Similar findings have been reported in pig production systems worldwide, where MDR *K. pneumoniae* has also been detected in swine (Mobasseri et al., 2019; Yang et al., 2019; Naing et al., 2025). Notably, four of these MDR isolates were identified as hypermucoviscous *K. pneumoniae*, a phenotype associated with excessive capsule production and commonly associated with hypervirulence (Dorman et al., 2018). The emergence of *K. pneumoniae* strains combining MDR and hypervirulent traits is particularly alarming due to their potential to cause invasive and difficult-to-treat infections, representing a significant challenge to public health (Kochan et al., 2023; Yang et al., 2024). Nevertheless, although these phenotypic findings suggest this convergence, a more comprehensive investigation of virulence-associated genes would be necessary for the proper characterization of hypervirulent strains.

Molecular detection of AMR genes was largely consistent with the phenotypic findings (Table 3), although the level of concordance varied among antimicrobial classes. Among tetracycline non-susceptible isolates, complete concordance was observed between the observed phenotype and the presence of the investigated *tet* genes, whereas quinolone and colistin non-susceptibility also showed high levels of genotype–phenotype agreement. In contrast, β-lactam resistance genes were detected more frequently than the corresponding phenotypic resistance. This discrepancy should be interpreted considering the study design, as β-lactam resistance genes were screened in all *Klebsiella* spp. isolates regardless of their susceptibility profile, whereas genes associated with the other antimicrobial classes were investigated only in phenotypically non-susceptible isolates. The presence of *bla* genes alone does not necessarily correlate with phenotypic β-lactam resistance, as the contribution of individual determinants depends on factors such as the specific β-lactamase variant, gene expression levels, and the presence of additional resistance mechanisms. Therefore, the detection of *bla* genes should be interpreted as an indication of resistance potential rather than as a direct predictor of the observed phenotype, particularly when multiple β-lactam agents and resistance determinants are evaluated simultaneously (Ellington et al., 2017).

The coexistence of multiple resistance determinants within the same isolates highlights the genetic complexity of AMR in *Klebsiella* spp. and supports previous reports describing the circulation of β-lactamase- and quinolone-associated resistance genes in swine-derived isolates (Zou et al., 2011; Mobasseri et al., 2019; Phetburom et al., 2021). This scenario may favor the maintenance and spread of AMR, potentially through horizontal gene transfer, particularly in the gastrointestinal tract, where dense bacterial populations facilitate genetic exchange (Huddleston, 2014; Ott and Mellata, 2022). The identification of multiple resistance genes within single isolates, including the coexistence of *bla*_SHV_, *bla*_TEM_, and *bla*_CTX–M_, suggests the potential emergence of extended-spectrum β-lactamase (ESBL)-producing *Klebsiella* spp. and underscores the need for continuous surveillance of these resistance determinants in swine production.

Genomic analysis expanded the phenotypic and PCR-based findings by showing that the four sequenced *K. pneumoniae* isolates from slaughtered pigs belonged to two sequence types with clinical relevance: ST45 and ST1027. The detection of ST45 is particularly noteworthy, as this lineage has been described among ESBL-producing *K. pneumoniae* associated with neonatal intensive care units and contaminated hospital environments (Shi et al., 2022), as well as in outbreaks involving colonized and infected pediatric patients (Fraser et al., 2025). In addition, GES-producing ST45 isolates have been reported in newborns and infants, reinforcing the clinical relevance and dissemination capacity of this lineage (Literacka et al., 2020).

In the present study, the ST45 isolates MSV_UFF_KB34 and MSV_UFF_KB38 carried 17 resistance genes distributed across ten antimicrobial classes, including *bla*_CTX–M–3_, *bla*_TEM–1D_, *qnrS1*, *aac(6*′*)-Ib-cr*, *sul1*, *sul2*, *tetA*, *mphA*, *floR*, *arr-3*, and *dfrA27*. The ST1027 isolates MSV_UFF_KB6 and MSV_UFF_KB21 also showed a broad resistome, including *bla*_CTX–M–15_, *bla*_TEM–1D_, *bla*_SHV–27_, *qnrS1*, *tetA*, *sul2*, *sul3*, *cmlA1*, *floR*, and aminoglycoside resistance genes. Public human-associated ST1027 genomes were scarce, and only one human ST1027 genome was retrieved and confirmed for comparison, suggesting that this lineage remains underrepresented in public genomic databases. Nevertheless, the detection of ST1027 in swine isolates carrying ESBL and other AMR genes highlights the need to expand genomic surveillance of *K. pneumoniae* from food-producing animals.

All four sequenced isolates carried virulence-associated loci related to iron acquisition, including yersiniabactin *ybt10* associated with *ICEKp4* and aerobactin *iuc3*, resulting in a Kleborate virulence score of 4. These findings indicate the presence of acquired siderophore-associated determinants. However, Kleborate scores should be interpreted as genomic surveillance metrics based on gene content rather than as direct predictors of clinical virulence. Although colibactin, salmochelin, and *rmpADC* were not detected, the coexistence of siderophore-associated virulence loci with ESBL and other AMR genes represents an epidemiologically relevant genomic feature. This is relevant because acquired virulence loci such as yersiniabactin and aerobactin are key markers used in genomic surveillance of *K. pneumoniae*, and AMR–virulence convergence has been increasingly recognized as a relevant public health threat (Lam et al., 2021). Further phenotypic and epidemiological studies are required to determine the biological relevance of these genomic findings.

Putative plasmid replicons were detected in all four sequenced MDR *K. pneumoniae* isolates, suggesting that plasmid-associated sequences may contribute to the genomic context of these isolates. However, these findings should be interpreted cautiously. Since only short-read sequencing was performed, complete plasmid reconstruction and definitive localization of antimicrobial resistance genes were not possible. Therefore, plasmid-mediated dissemination and horizontal gene transfer should be considered plausible mechanisms rather than processes directly demonstrated by the present study.

The findings of this study should be interpreted considering some limitations. Whole-genome sequencing was conducted on four MDR *K. pneumoniae* isolates selected based on their antimicrobial resistance profiles, providing insights into the genomic characteristics of representative strains identified in this study. However, this selection strategy was not designed to capture the full genomic diversity of *K. pneumoniae* circulating in slaughter pigs in Brazil. Therefore, the identification of specific sequence types (ST45 and ST1027), K/O antigen loci, and virulence-associated loci, such as *ybt10*/ICEKp4 and *iuc3*, should be interpreted as characteristics of the sequenced isolates rather than as population-level findings. Larger genomic studies including a broader and more representative collection of isolates from different farms, regions, and production systems are required to better assess the frequency and dissemination of these genomic profiles in swine production. Additionally, genomic analyzes were performed using short-read sequencing, which limits the resolution required to determine the precise genomic context and location of antimicrobial resistance genes, particularly regarding their association with plasmids and other mobile genetic elements (Ellington et al., 2017). Although mobile genetic elements play an important role in the dissemination of these determinants in *K. pneumoniae*, long-read sequencing approaches would provide further insights into plasmid structures and the potential mechanisms involved in horizontal gene transfer. Finally, the colistin-resistant isolate lacking the investigated *mcr* genes warrants further investigation to determine the genetic basis of this phenotype, including the potential contribution of alternative chromosomal mutations or resistance determinants not evaluated in this study.

Overall, the results reinforce the relevance of swine production systems in the epidemiology of AMR within the One Health perspective. Intensive production systems, particularly those involving repeated antimicrobial exposure, may act as amplification points for resistant bacteria and resistance determinants, potentially contributing to their dissemination across different ecological niches, including the food production chain (Conceição et al., 2023; Oyenuga et al., 2024).

## Data Availability

The datasets presented in this study can be found in online repositories. The names of the repository/repositories and accession number(s) can be found in the article/Supplementary material.

## Footnotes

1
https://github.com/ablab/spades

2
https://github.com/klebgenomics/Kleborate

3
https://github.com/marbl/Mash
