# Supplementary material for: Slaughtered pigs as reservoirs of One Health-relevant antimicrobial resistance genes in Klebsiella pneumoniae from Brazil
Source: Front Microbiol. 2026 Sep 9;17:1927798. doi: 10.3389/fmicb.2026.1927798 (PMC13597807; doi:10.3389/fmicb.2026.1927798)
Supplement: Supplementary file 1 [file Table_1.docx]

Supplementary Material

**Table S1**. Primers used for the identification of *Klebsiella* spp. and differentiation of species.

| Target gene | Target | Primer sequence (5′–3′) | Amplicon size (bp) | Annealing temperature (°C) | Reference |
| --- | --- | --- | --- | --- | --- |
| *gyrA* | *Klebsiella* spp. | F-CGCGTACTATACGCCATGAACGTA  R-ACCGTTGATCACTTCGGTCAGG | 441 | 50 °C | (Brisse and Verhoef, 2001) |
| *bla*_SHV_ | *K. pneumoniae* | F-GCTGGCGGTACACGCCAGCCCG  R-AGAAGCATCCTGCTGTGCG | 995 | 55 °C | (Fonseca et al., 2017) |
| *bla*_LEN_ | *K. variicola* | F-CACGCTGCGYAAACTACTGACYGCGCAGCA  R-AGAAGCATCCTGCTGTGCG | 485 |  | (Fonseca et al., 2017) |
| *bla*_OKP_ | *K. quasipneumoniae* | F-GGCCGGYGAGCGGGGCTCA  R-AGAAGCATCCTGCTGTGCG | 348 |  | (Fonseca et al., 2017) |
| *pehX* | *K. oxytoca* | F- GATACGGAGTATGCCTTTACGGTG  R- TAGCCTTTATCAAGCGGATACTGG | 344 | 59 °C | (Kovtunovych et al., 2003) |

**Table S2**. Primers used for the detection of antimicrobial resistance genes in *Klebsiella* spp. isolates.

| Antimicrobial class | Targetgene | Primer sequence (5′–3′) | Amplicon size (bp) | Annealing temperature (°C) | Reference |
| --- | --- | --- | --- | --- | --- |
| Colistin | *mcr-1* | F- AGTCCGTTTGTTCTTGTGGC  R- AGATCCTTGGTCTCGGCTTG | 320 | 58 °C | (Rebelo et al., 2018) |
|  | *mcr-2* | F- CAAGTGTGTTGGTCGCAGTT  R- TCTAGCCCGACAAGCATACC | 715 |  |  |
|  | *mcr-3* | F- AAATAAAAATTGTTCCGCTTATG  R- AATGGAGATCCCCGTTTTT | 929 |  |  |
|  | *mcr-4* | F- TCACTTTCATCACTGCGTTG  R- TTGGTCCATGACTACCAATG | 1,116 |  |  |
|  | *mcr-5* | F- ATGCGGTTGTCTGCATTTATC  R- TCATTGTGGTTGTCCTTTTCTG | 1,644 |  |  |
|  | *mcr-6* | F- AGCTATGTCAATCCCGTGAT  R- ATTGGCTAGGTTGTCAATC | 252 | 55 °C | (Borowiak et al., 2020) |
|  | *mcr-7* | F- GCCCTTCTTTTCGTTGTT  R- GGTTGGTCTCTTTCTCGT | 551 |  |  |
|  | *mcr-8* | F- TCAACAATTCTACAAAGCGTG  R- AATGCTGCGCGAATGAAG | 856 |  |  |
|  | *mcr-9* | F- TTCCCTTTGTTCTGGTTG  R- GCAGGTAATAAGTCGGTC | 1011 |  |  |
|  | *mcr-10* | F- GGACCGACCTATTACCAGCG  R- GGCATTATGCTGCAGACACG | 366 | 64 °C | (Lei et al., 2020) |
| β-lactams | *bla*_SHV_ | F- ATGCGTTATATTCGCCTGTG  R- TGCTTTGTTATTCGGGCCAA | 747 | 60 °C | (Monstein et al., 2007) |
|  | *bla*_TEM_ | F- TCGCCGCATACACTATTCTCAGAATGA  R- ACGCTCACCGGCTCCAGATTTAT | 445 |  |  |
|  | *bla*_CTX-M_ | F- ATGTGCAGYACCAGTAARGTKATGGC  R- TGGGTRAARTARGTSACCAGAAYCAGCGG | 593 |  |  |
|  | *bla*_CMY_ | F-ATGATGAAAAAATCGTTATGC  R-TTGCAGCTTTTCAAGAATGCGC | 1143 | 65 °C | (Koeck et al., 2006) |
| Quinolones | *qnrA* | F- AGAGGATTTCTCACGCCAGG  R- TGCCAGGCACAGATCTTGAC | 580 | 58 °C | (Kraychete et al., 2016) |
|  | *qnrB* | F- GGMATHGAAATTCGCCACTG  R- TTTGCYGYYCGCCAGTCGAA | 264 |  |  |
|  | *qnrC* | F- GCGAATTTCCAAGGGGCAAA  R- ACCCGTAATGTAAGCAGAGCAA | 135 |  |  |
|  | *qnrD* | F- AGGTGTAGCATGTATGGAAAAGC  R- ACATTGGGGCATTAGGCGTT | 691 |  |  |
|  | *qnrS* | F- GCAAGTTCATTGAACAGGGT  R- TCTAAACCGTCGAGTTCGGCG | 428 |  |  |
|  | *oqxAB* | F- GACAGCGTCGCACAGAATG  R- GGAGACGAGGTTGGTATGGA | 339 | 65 °C | (Chen et al., 2012) |
|  | *aac(6′)-Ib-cr* | F- TTGCGATGCTCTATGAGTGGCTA  R- CTCGAATGCCTGGCGTGTTT | 482 | 60 °C | (Park et al., 2006) |
|  | *qepA* | F- GCAGGTCCAGCAGCGGGTAG  R-CTTCCTGCCCGAGTATCGTG | 199 | 60 °C | (Yamane et al., 2008) |
| Tetracyclines | *tetA* | F- GCTACATCCTGCTTGCCTTC  R- CATAGATCGCCGTGAAGAGG | 210 | 55°C | (Ng et al., 2001) |
|  | *tetB* | F- TTGGTTAGGGGCAAGTTTTG  R- GTAATGGGCCAATAACACCG | 659 |  |  |
|  | *tetC* | F- CTTGAGAGCCTTCAACCCAG  R- ATGGTCGTCATCTACCTGCC | 418 |  |  |
|  | *tetD* | F- AAACCATTACGGCATTCTGC  R- GACCGGATACACCATCCATC | 787 |  |  |
|  | *tetE* | F- AAACCACATCCTCCATACGC  R- AAATAGGCCACAACCGTCAG | 278 |  |  |
|  | *tetG* | F- GCTCGGTGGTATCTCTGCTC  R- AGCAACAGAATCGGGAACAC | 468 |  |  |

**Table S3**. Sequencing quality metrics and genome assembly statistics for the four *Klebsiella pneumoniae* isolates included in the whole-genome analysis.

| Isolate | BioSample | ST | Read pairs | Bases (Gb) | Q20 (%) | Q30 (%) | Raw GC (%) | Contigs | Genome size (bp) | Assembly GC (%) | N50 (bp) | N90 (bp) | Largest contig (bp) | Mean depth |
| --- | --- | --- | --- | --- | --- | --- | --- | --- | --- | --- | --- | --- | --- | --- |
| MSV_UFF_KB6 | SAMN61231285 | ST1027 | 10,438,956 | 3.13 | 99.01 | 95.15 | 57.31 | 103 | 5,681,264 | 56.98 | 272,054 | 46,095 | 626,221 | 265.41 |
| MSV_UFF_KB21 | SAMN61231286 | ST1027 | 9,927,759 | 2.98 | 99.02 | 95.28 | 57.13 | 73 | 5,433,734 | 57.24 | 233,891 | 45,665 | 768,248 | 274.28 |
| MSV_UFF_KB34 | SAMN61231287 | ST45 | 10,219,116 | 3.06 | 99.01 | 95.19 | 57.31 | 115 | 5,670,243 | 57.02 | 296,421 | 86,340 | 813,974 | 274.09 |
| MSV_UFF_KB38 | SAMN61231288 | ST45 | 11,078,435 | 3.32 | 99.07 | 95.40 | 57.27 | 124 | 5,723,483 | 56.98 | 296,421 | 77,074 | 1,017,239 | 549.41 |

The whole-genome sequencing data generated in this study have been deposited in the NCBI database under BioProject accession number PRJNA1484406.

**Table S4**. Plasmid replicons detected among sequenced *Klebsiella pneumoniae* isolates.

| Isolate | BioSample | Replicon | Contig | Identity_percent | Coverage_percent | Alignment_length | Replicon_length | Start | End | Evalue | Bitscore |
| --- | --- | --- | --- | --- | --- | --- | --- | --- | --- | --- | --- |
| MSV_UFF_KB6 | SAMN61231285 | IncFII(K)_1__CP000648 | KB6_contig_34 | 98,00 | 100,00 | 148 | 148 | 12821 | 12674 | 3.86e-71 | 263 |
| MSV_UFF_KB6 | SAMN61231285 | IncFIB(AP001918)_1__AP001918 | KB6_contig_43 | 98,00 | 100,00 | 682 | 682 | 1759 | 1078 | 0.0 | 1188 |
| MSV_UFF_KB6 | SAMN61231285 | IncFIB(pKPHS1)_1__CP003223 | KB6_contig_16 | 99,00 | 100,00 | 560 | 560 | 77121 | 77680 | 0.0 | 1024 |
| MSV_UFF_KB6 | SAMN61231285 | ColRNAI_1__DQ298019 | KB6_contig_36 | 97,00 | 100,00 | 130 | 130 | 6261 | 6390 | 1.57e-59 | 224 |
| MSV_UFF_KB6 | SAMN61231285 | repB(R1701)_1__CP039970 | KB6_contig_22 | 99,00 | 100,00 | 623 | 623 | 13202 | 13824 | 0.0 | 1123 |
| MSV_UFF_KB21 | SAMN61231286 | IncFII(K)_1__CP000648 | KB21_contig_34 | 98,00 | 100,00 | 148 | 148 | 12821 | 12674 | 3.86e-71 | 263 |
| MSV_UFF_KB21 | SAMN61231286 | IncFIB(AP001918)_1__AP001918 | KB21_contig_43 | 98,00 | 100,00 | 682 | 682 | 1759 | 1078 | 0.0 | 1188 |
| MSV_UFF_KB21 | SAMN61231286 | IncFIB(pKPHS1)_1__CP003223 | KB21_contig_16 | 99,00 | 100,00 | 560 | 560 | 77121 | 77680 | 0.0 | 1024 |
| MSV_UFF_KB21 | SAMN61231286 | ColRNAI_1__DQ298019 | KB21_contig_36 | 97,00 | 100,00 | 130 | 130 | 6261 | 6390 | 1.57e-59 | 224 |
| MSV_UFF_KB21 | SAMN61231286 | repB(R1701)_1__CP039970 | KB21_contig_22 | 99,00 | 100,00 | 623 | 623 | 13202 | 13824 | 0.0 | 1123 |
| MSV_UFF_KB34 | SAMN61231287 | IncFII(K)_1__CP000648 | KB_34_contig_33 | 98,00 | 100,00 | 148 | 148 | 6370 | 6223 | 3.86e-71 | 263 |
| MSV_UFF_KB34 | SAMN61231287 | IncFIB(K)_1__JN233704 | KB_34_contig_20 | 99,00 | 100,00 | 560 | 560 | 78187 | 78746 | 0.0 | 1024 |
| MSV_UFF_KB34 | SAMN61231287 | IncQ1_1__M28829 | KB_34_contig_39 | 100,00 | 65,70 | 523 | 796 | 523 | 1 | 0.0 | 966 |
| MSV_UFF_KB34 | SAMN61231287 | ColRNAI_1__DQ298019 | KB_34_contig_34 | 97,00 | 100,00 | 130 | 130 | 5986 | 6115 | 1.56e-59 | 224 |
| MSV_UFF_KB34 | SAMN61231287 | repB(R1701)_1__CP039970 | KB_34_contig_28 | 99,00 | 100,00 | 623 | 623 | 2151 | 2773 | 0.0 | 1123 |
| MSV_UFF_KB38 | SAMN61231288 | IncFII(K)_1__CP000648 | KB_38_contig_37 | 98,00 | 100,00 | 148 | 148 | 6370 | 6223 | 3.86e-71 | 263 |
| MSV_UFF_KB38 | SAMN61231288 | IncFIB(K)_1__JN233704 | KB_38_contig_20 | 99,00 | 100,00 | 560 | 560 | 78187 | 78746 | 0.0 | 1024 |
| MSV_UFF_KB38 | SAMN61231288 | IncQ1_1__M28829 | KB_38_contig_42 | 100,00 | 65,83 | 524 | 796 | 3864 | 4387 | 0.0 | 968 |
| MSV_UFF_KB38 | SAMN61231288 | ColRNAI_1__DQ298019 | KB_38_contig_38 | 97,00 | 100,00 | 130 | 130 | 5986 | 6115 | 1.58e-59 | 224 |
| MSV_UFF_KB38 | SAMN61231288 | repB(R1701)_1__CP039970 | KB_38_contig_31 | 99,00 | 100,00 | 623 | 623 | 12568 | 11946 | 0.0 | 1123 |

The whole-genome sequencing data generated in this study have been deposited in the NCBI database under BioProject accession number PRJNA1484406.
